# Chromosome transplantation as a novel approach for correcting complex genomic disorders

**DOI:** 10.18632/oncotarget.6143

**Published:** 2015-10-17

**Authors:** Marianna Paulis, Alessandra Castelli, Lucia Susani, Michela Lizier, Irina Lagutina, Maria Luisa Focarelli, Camilla Recordati, Paolo Uva, Francesca Faggioli, Tui Neri, Eugenio Scanziani, Cesare Galli, Franco Lucchini, Anna Villa, Paolo Vezzoni

**Affiliations:** ^1^ Milan Unit, Istituto di Ricerca Genetica e Biomedica, CNR, Milan, Italy; ^2^ Humanitas Clinical and Research Center, Rozzano, Milan, Italy; ^3^ Centro Ricerche Biotecnologiche, Università Cattolica del Sacro Cuore, Cremona, Italy; ^4^ Avantea, Cremona, Italy; ^5^ Mouse and Animal Pathology Laboratory, Fondazione Filarete, Milan, Italy; ^6^ CRS4 Bioinformatics Laboratory, Parco Scientifico e Tecnologico POLARIS, Pula, Cagliari, Italy; ^7^ Department of Veterinary Sciences and Public Health, University of Milan, Milan, Italy; ^8^ Department of Veterinary Medical Sciences, University of Bologna, Ozzano Emilia, Bologna, Italy

**Keywords:** embryonic stem cells, chromosome transplantation, microcell fusion, genomic disorders, cell therapy

## Abstract

Genomic disorders resulting from large rearrangements of the genome remain an important unsolved issue in gene therapy. Chromosome transplantation, defined as the perfect replacement of an endogenous chromosome with a homologous one, has the potential of curing this kind of disorders. Here we report the first successful case of chromosome transplantation by replacement of an endogenous X chromosome carrying a mutation in the *Hprt* gene with a normal one in mouse embryonic stem cells (ESCs), correcting the genetic defect. The defect was also corrected by replacing the Y chromosome with an X chromosome. Chromosome transplanted clones maintained *in vitro* and *in vivo* features of stemness and contributed to chimera formation. Genome integrity was confirmed by cytogenetic and molecular genome analysis. The approach here proposed, with some modifications, might be used to cure various disorders due to other X chromosome aberrations in induced pluripotent stem (iPS) cells derived from affected patients.

## INTRODUCTION

The hope of correcting genetic diseases has been around for long time, since the first gene transfer to cultured cells was documented [1]. Although very simple in theory, gene therapy has met both expected and unexpected difficulties, regarding inefficient gene transfer, random integration, silencing of the transferred gene and difficult expansion and differentiation of the specific cell types needed to rescue the phenotype.

These limits apply to essentially any genetic disease to be studied. However, a few genetic defects show an additional difficulty. We refer here to those that can be listed as “genomic disorders” [2], which apparently could neither be treated with conventional techniques such as viral vectors, nor are likely to be treated with the homologous recombination strategy. Among these are any “structural” abnormalities, which include large deletions or inversions, copy number variations (CNV) and complex rearrangements. Any new approach aimed at solving the specific hurdles presented by these genomic disorders will be of interest.

Chromosome transplantation can be defined as the perfect replacement of an endogenous chromosome with a homologous one, resulting in a normal diploid cell. However, since the chromosome transfer process cannot be tightly controlled, the resulting cells usually bear an abnormal content of chromosomes and are therefore aneuploid. These cells cannot be used to obtain normal organisms and are of little value for therapeutic aims [3]. Microcell mediated chromosome transfer (MMCT) allows the transfer of a single chromosome to a recipient cell [4, 5]. With this technique a single exogenous minichromosome has been added not only to several neoplastic cell lines, but also to mouse pluripotent stem cells [6, 7]. Notably, by blastocyst injection of mouse ESC in which a human chromosome fragment has been transferred by MMCT, fertile “transchromosomal” chimeric mice have been generated [8]. Transchromosomal calves have also been obtained by nuclear transfer of bovine fibroblasts where an artificial human chromosome was transferred by MMCT [9].

Here we tested the possibility of transferring a single exogenous chromosome and subsequently eliminating its endogenous mutated homologue. Microcell-based approaches have been used to transfer single chromosomes in various types of cells and tools to eliminate an endogenous chromosome have also been proposed [10, 11], although cells with a normal diploid genome content after chromosome substitution has, to our knowledge, never been clearly demonstrated. Focusing on the sex chromosomes as the best candidates for this purpose, we set up a two-step plan, based on isolation of clones with a single additional X chromosome followed by selection of clones that lost one of the endogenous sex chromosomes.

## RESULTS

### The experimental plan

**Figure 1:**
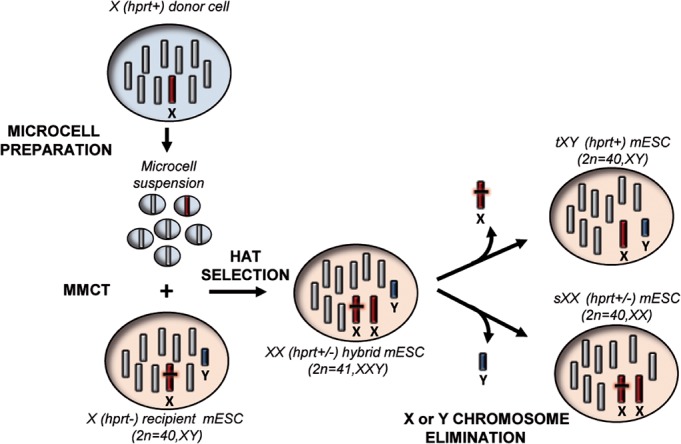
Schematic overview of the “genomic therapy” approach The scheme shows the step by step procedure followed to generate mouse ESCs in which an endogenous chromosome is replaced with an exogenous one. *Hprt*: Hypoxanthine phosphoribosyltransferase, MMCT: Microcell mediated chromosome transfer, HAT medium: hypoxanthine-aminopterin-thymidine medium, tXY: transplanted XY mouse ESCs, sXX: substituted XX mouse ESCs.

The procedure envisaged to generate mouse ESCs in which an endogenous chromosome is exchanged with an exogenous one is outlined in Figure 1. In the first step, an exogenous normal mouse X chromosome (*Hprt*+) was transferred through MMCT into recipient male mouse ESCs carrying a mutation in the *Hprt* gene (*Hprt*-). The resulting cells were selected in HAT medium, to identify those in which the normal X chromosome has been acquired. In the second step, clones in which an endogenous sex chromosome has been lost were identified from the initial pool; these clones could become either transplanted XY (tXY), resulting from loss of the endogenous X chromosome, or, alternatively, substituted XX (sXX) resulting from loss of the endogenous Y chromosome.

### Isolation and characterization of transplanted E14TG2a XY clones

In order to obtain a donor cell line with a normal X chromosome suitable for MMCT, we fused the mouse A9 cells (*Hprt* defective) with MEF obtained from a pool of CD1 embryos, an outbred strain, and then selected a hybrid clone (A9/MEF-C12) with a normal MEF-derived X chromosome. This step was necessary since normal MEF are not suitable for micronucleation, while the A9 cell line has been widely used for this purpose [12]. As recipient cell line, we used the male E14TG2a ESC line derived from the 129/Ola strain. Microcells were obtained from the donor A9/MEF-C12 and fused with E14TG2a cells. Resulting fused cells were selected in HAT medium to identify clones which became *Hprt+*. In the first successful MMCT experiment we obtained two HAT-resistant clones (clone 1 and clone 5), both with a 41,XXY karyotype as demonstrated by chromosome number analysis and FISH experiments using a whole mouse X chromosome painting and an X-linked BAC as probes (Figures 1a and 1b Supplemental). Karyotype analysis of both clones revealed XXY and XY cells when further cultured in non-selective medium. Several XY and XXY subclones were then isolated and selected again in HAT medium, but unfortunately, while the XXY subclones were resistant, all the XY subclones were HAT-sensitive (data not shown). This means that only the exogenous X chromosome (*Hprt*+) was lost when the HAT selection was relieved. Therefore, we were unable to isolate clones with transplanted X chromosomes.

**Figure 2:**
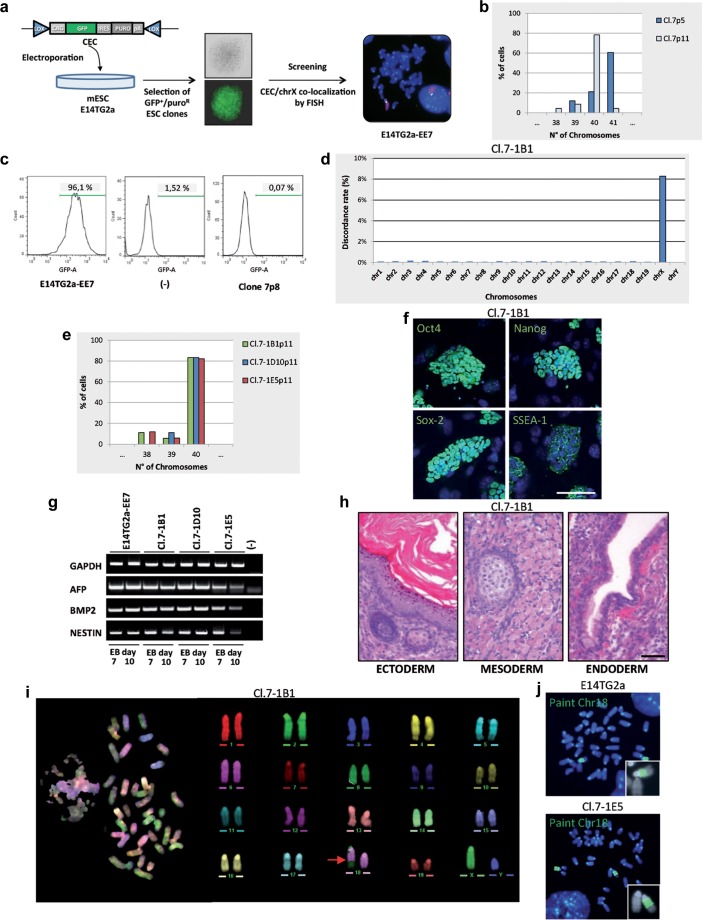
Isolation and characterization of the tXY clones **a.** Schematic diagram showing the isolation of E14TG2a clones carrying the CEC plasmid on the X chromosome. Whole X chromosome painting (red) and CEC vector (green) were used as probes in metaphase FISH. CEC, chromosome elimination cassette vector: puro, puromycine. **b.** Chromosome number distribution (*n* = 30 each). **c.** Flow cytometry analysis of GFP expression. **d.** Chromosome distribution of the SNP diversity. Cl.7-1B1 is compared to the E14TG2a parental cell line. **e.** Chromosome number distribution of the 3 subclones (*n* = 30 each). **f.** Immunostaining for stemness markers (green). Scale bar, 100 μm. **g.** mRNA expression analysis of the differentiation markers on day 7 and 10 embryoid bodies (EB day 7 and EB day 10) by RT-PCR. GAPDH is used as control. **h.** Teratoma formation assay. H&E stainings show keratinized stratified squamous epithelium (ectoderm), skeletal muscle and focus of early cartilaginous differentiation (mesoderm), columnar ciliated epithelium with goblet cells (endoderm). Scale bar, 50 μm. **i.** M-FISH metaphase spread and corresponding pseudo-colored karyotype. The red arrow indicates the rearrangement. **j.** Metaphases FISH; whole mouse chromosome 18 painting (green) was used as probe. All passage numbers are indicated with p.

In view of these results, and in order to facilitate the loss of the endogenous X chromosome, we tried the approach described by Matsumura and coworkers who devised a chromosome elimination cassette (CEC) bearing the *gfp* reporter gene and the puromycin gene flanked by two oppositely oriented loxP sites [10]. This vector was specifically designed in order to allow the elimination of a targeted chromosome. We transfected the E14TG2a line with the CEC plasmid, selected puromycin resistant GFP^+^ clones, and identified by FISH analysis a clone (E14TG2a-EE7) in which the plasmid had integrated onto the X chromosome (Figure 2a). The MMCT procedure was repeated and a single HAT-resistant clone (clone 7) with only one single additional X chromosome (41,XXY) was isolated. Chromosome number analysis of the expanded clone showed that after a few passages, the percentage of 41,XXY cells spontaneously dramatically decreased (Figure 2b); moreover, flow cytometry analysis revealed the loss of the GFP positivity (Figure 2c). This suggested that the cells lost the endogenous X chromosome (*gfp+*/*Hprt*-) but retained the exogenous X chromosome (*Hprt*+). For this reason, the elimination strategy based on the CEC vector became unnecessary; therefore it was not performed and 33 subclones were directly isolated.

To unequivocally demonstrate that the cells had replaced the “defective” endogenous chromosome with a normal one, we amplified the *Hprt* locus to discriminate the mutated from the *wild type* (*wt*) sequence [13]. In the presence of the E14TG2a deletion, which encompasses exons 1 and 2, a 628 bp-fragment was amplified, while the *wt* locus gave a 746 bp-amplicon. As expected, both bands were evident in the initial XXY population (clone 7), while only the *wt* band appeared in all the 33 clones (Figure 2a Supplemental). In addition to verify the chromosome replacement, we identified four X-chromosome specific polymorphic loci which discriminate between the 129/Ola (E14TG2a) and the CD1 (MEF) genome. PCR and sequence analysis of these SNPs performed on 3 tXY clones (cl.7-1B1, cl.7-1D10, cl.7-1E5) showed only the CD1 variants, while the XXY colony (clone 7) showed both the 129/Ola and the CD1 ones (Figure 2b Supplemental). Taken together, these data indicate that the tXY clones had indeed exchanged the endogenous X chromosome with the exogenous one.

To further support this conclusion and to globally evaluate whether the complex cell manipulations altered the whole genome structure of the subclones, we performed an SNP and CNV analyses by microarray, using the platform provided by The Jackson Laboratories (see Material and methods section). The parental E14TG2a cells and three tXY subclones were analyzed. As expected, SNPs mapping to the 19 autosomes and the Y chromosome were virtually identical between the parental cell line E14TG2a (129/Ola strain) and the three clones (Figure 2d). Furthermore, the CNV analysis performed on all the autosomes did not detect any copy number alterations between the clones and the parental cell line. On the contrary, SNP analysis of the X chromosome detected several differences between the parental cells and the three subclone, as expected from the substitution of an X chromosome on the 129/Ola strain with one deriving from a different outbred strain (CD1). Taken together, these findings clearly demonstrate that the substitution of an individual mouse chromosome can be achieved by our experimental approach by simply exploiting the spontaneous loss of a supernumerary chromosome and that the chromosome transplantation procedure did not appreciably affect the genome integrity of the resulting cell.

Moreover, cytogenetic analysis revealed that the three tXY subclones showed a normal mouse complement of 40 chromosomes (Figure 2e). They were also analysed to determine their pluripotency, and the results obtained on the Cl.7-1B1 subclone are shown in Figures 2f-2h. Immunofluorescence analysis of the Oct4, Nanog, Sox2 and SSEA-1 pluripotent markers showed that these genes were expressed (Figure 2f). Similar results were obtained by Real-Time PCR (data not shown). To demonstrate functional pluripotency *in vitro*, the subclones were allowed to differentiate into embryoid bodies and analyzed the expression of lineage specific genes (Figure 2g). Finally, the clones injected into nude mice gave rise to teratomas (Figure 2h). Altogether these results show that the E14TG2a-derived tXY subclones maintained pluripotency.

Unfortunately, detailed analysis of the tXY subclones by standard banding analysis and by multicolor FISH (M-FISH) showed a small rearrangement involving the chromosome 18 (Figure 2i) in all of them as well as in the parental cell line before microcell fusion. This rearrangement was confirmed by FISH experiments using the whole chromosome 18 painting as probe (Figure 2j). Despite this observation, two of these clones were injected into blastocysts but no chimeric mice were obtained. We did not further investigate whether this inability to produce chimeric mice was due to the rearrangement itself or to all the manipulations that the cells underwent during the procedure. Of note, the E14TG2a cell line is reported to colonize the germline with a low efficiency [14].

### Isolation and characterization of substituted HM1 XX clones

**Figure 3:**
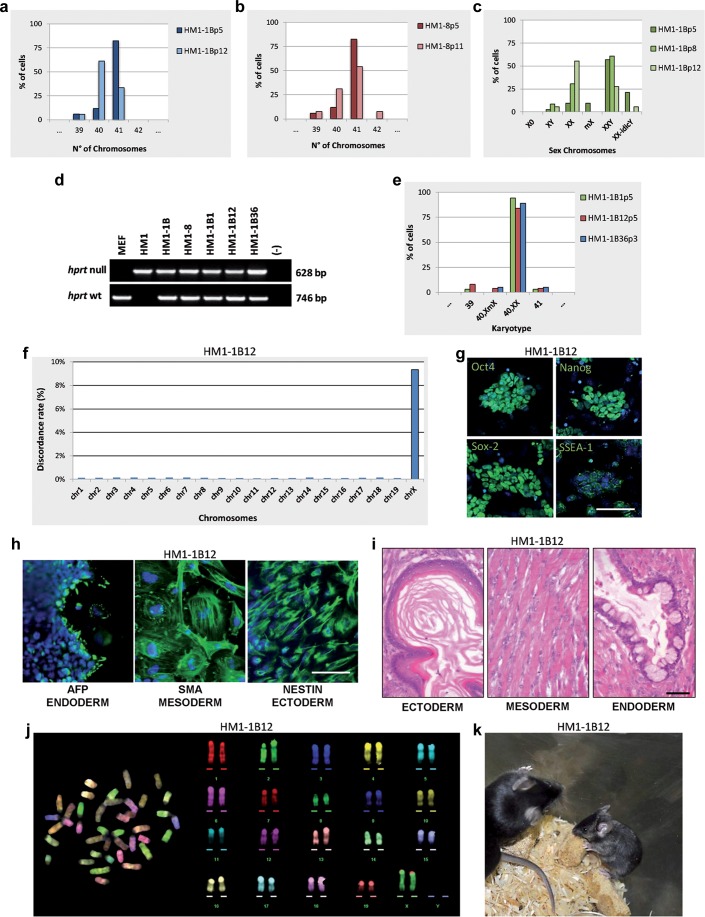
Isolation and characterization of the sXX clones **a.**, **b.** Chromosome number distributions (*n* = 30 each). **c.** Sex chromosome analysis by FISH using the whole mouse X and Y chromosome painting probes. **d.** Genomic PCR of *Hprt* gene on indicated clones. **e.** Karyotype distribution (*n* = 30 each). **f.** Chromosome distribution of the SNP diversity. HM1-1B12 clone is compared to the HM1 parental cell line. **g.** Immunostaining for stemness markers (green). Scale bar, 100 μm. **h.** Immunostaining for embryoid bodies (EB). Scale bar, 50 μm. **i.** Teratoma formation assay. H&E stainings show keratinized stratified squamous epithelium (ectoderm), skeletal muscle (mesoderm), columnar ciliated epithelium with goblet cells, (endoderm). Scale bar, 50 μm. **j.** M-FISH metaphase spread and corresponding pseudo-colored karyotype. **k.** Chimaeric mouse generated by a sXX clone. All passage numbers are indicated with p.

E14TG2a cells were among the first mouse ESC lines produced [15]. They were a subclone obtained by selection of E14 ESC line in 6-thyoguanine, a procedure allowing the isolation of *Hprt* defective cells. These cells were also used to generate mice from which a new *Hprt-* ESC, the HM1 line, was derived from the blastocysts. HM1 has been reported to be quite efficient in germline colonization [14]. Therefore, to have a better chance of obtaining mouse chimeras, we switched to the HM1 line, on which identical MMCT experiments were performed. Two clones (HM1-1B and HM1-8) growing in HAT medium showed in the first passages in culture to be mainly composed of 41 chromosomes. FISH and karyotype analysis at subsequent passages started showing a large fraction of 40,XX cells, in addition to a few XY cells and some cells with a rearrangement of both sex chromosomes (Figure 3a-3c and Figure 3 Supplemental). Subcloning of one of the two original clones (HM1-1B) led to the isolation of several sXX subclones with a normal mouse 40,XX karyotype, although we were unable to isolate any XY clone. Confirmation was obtained on selected subclones (HM1-1B1, HM1-1B12 and HM1-1B36) by PCR, karyotype and FISH using the whole X and Y chromosome painting probes (Figure 3d, 3e and Figures 3b,3c Supplemental).

Furthermore, mouse diversity array analysis and comparison of the three sXX subclones with the parental HM1 cell line, showed an increased level of SNP discordance only at the X chromosome level (Figure 3f); moreover, no Y chromosome SNPs were detected in the sXX subclones. Both SNP and CNV analysis were virtually identical for all the autosomes. Taken together, these findings strongly suggest that the endogenous Y chromosome has been substituted with the exogenous X chromosome, thus giving rise to a normal female cell (40,XX, *Hprt*+/−) and confirms the previous finding that the procedure allows the substitution of an entire normal chromosome without affecting the genome integrity of the cells.

The selected sXX subclones were also analyzed for markers of pluripotency and differentiation capacity. Real-Time PCR expression analysis (data not shown), immunofluorescence analysis of stemness genes, *in vitro* differentiation into the three germ layers and teratoma formation suggested that the three subclones maintained the original pluripotency. The analyses performed on the HM1-1B12 clone are shown in Figures 3g-3i. The karyotypically normal HM1-1B12 (Figure 3j) and HM1-1B36 subclones were used for further experiments *in vivo*, after having assessed the efficiency of the HM1 cell line in generating chimeras and in germline colonization. By blastocyst injections we obtained 3 fertile chimeras for both clones: 2 males and 1 female for HM1-1B12 and 1 male and 2 females for HM1-1B36, respectively. However, all the females were low-grade chimeras and the one derived from HM1-1B12 (Figure 3k) appeared smaller than normal. Chimeric females were fertile but none of them gave rise to offsprings deriving from the sXX subclones. XX cells in male blastocysts cannot form gametes.

### Isolation and characterization of “rejuvenated” ESC lines

**Figure 4:**
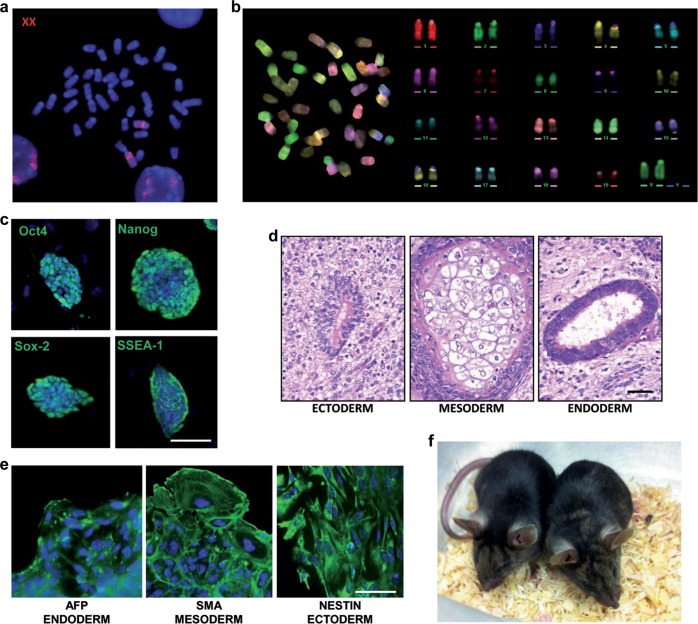
Characterization of the NT-E3.2 rejuvenated clone **a.** FISH metaphase spread. Whole mouse X chromosome painting (red) was used as probe. **b.** M-FISH metaphase spread and corresponding pseudo-colored karyotype. **c.** Immunostaining for stemness markers (green). Scale bar, 100 μm. **d.** Teratoma formation assay. H&E stainings show mature nervous tissue surrounding a rosette of primitive neuroepithelium (ectoderm), cartilage (mesoderm), a glandular structure lined by a columnar ciliated epithelium (endoderm), Scale bar, 50 μm. **e.** Immunostaining for embryoid bodies (green), scale bar, 50 μm. **f.** Chimaeric mice generated by an NT-clone.

XX ESCs are somewhat more difficult to handle than XY ESCs, probably due to the fact that X chromosome gene dosage is tightly controlled in early embryos [16]. Therefore, in order to reprogram the normal X chromosome dosage in the oocyte environment, we performed nuclear transfer (NT) in mouse oocytes with HM1-1B12 cells as donors. The nuclear transfer blastocysts that developed were plated and 3/27 of these “rejuvenated” ESC lines (NT-clones) with normal mouse 40,XX karyotype were obtained. Molecular PCR analysis of both forms of the *Hprt* loci (*wt* and deleted) and of selected X-linked SNPs confirmed the presence of the two different X chromosomes (Figures 4a, 4b Supplemental). Karyotype stability analysis during *in vitro* culture revealed that the three NT-clones acquired a greater stability of the X chromosome; unlike the original HM1-1B12 parental clone (Figure 5a Supplemental), rearrangements involving the X chromosome were not observed (Figures 5b-5d Supplemental). One of these NT-clones (Clone NT-E3.2) was further characterized by FISH using X chromosome painting as probe, M-FISH, stemness marker expression, and *in vivo* and *in vitro* differentiation capacity (Figures 4a-4e). This clone maintained a correct karyotype up to passage 10 with two X chromosomes. This clone was injected into C57BL/6N blastocysts and 2 female and 1 male fertile chimeras with a normal phenotype were obtained (Figure 4f).

## DISCUSSION

In spite of the widespread use of cell hybridization techniques, and the repeated demonstration that both complementation of genetic defects and reprogramming can be achieved by cell fusion, the inability to control the number of transferred chromosomes and the deriving aneuploidy status has so far prevented its application in investigating important issues in biology as well as its exploitation for therapeutic aims. To obtain hybridization products with normal diploid content, techniques allowing for limited transfer of genetic material, such as MMCT can be useful; in this regard, several reports of a single chromosome transfer have long been present in literature, although most of them have not been carefully characterized with cytogenetic and molecular techniques in order to show concomitant undesirable transfer of extra DNA fragments. However, procedures to eliminate the endogenous homologous chromosome have not yet been standardized, precluding the generation of cells in which an endogenous chromosome has been substituted with a normal one.

To eliminate the supernumerary chromosome, two main approaches can be followed. The first is the isolation of clones that spontaneously lose the additional chromosome. This has been shown to occur in nature and even in human pathology, due to the existence of “mosaic” individuals bearing both trisomic and disomic cell populations [17]. The identification of these clones can be achieved by engineering the targeted chromosome with genes that can be selected against, such as the thymidine kinase (TK) gene. Recently, Li and coworkers engineered a chromosome 21 in an induced pluripotent stem (iPS) cell line from a patient affected by Down syndrome with a TK carrying plasmid and obtained a large percentage of diploid clones after exposure to gancyclovir [11]. However, the same result was obtained simply analyzing clones of trisomic cells grown *in vitro* [18]. In our three successful experiments in which a single chromosome was transferred to ESC lines, diploid clones (either XY or XX) were easily isolated, although we were unable to control the specific extra-chromosome loss. Moreover, in the first case, only the exogenous X chromosome was lost, in the second only the endogenous one, whereas in the third the endogenous Y was substituted by an exogenous X chromosome. In the last two cases, diploid cells, in which the *Hprt* defect was rescued, were obtained.

In the second approach an active removal of the chromosome by exploiting cre-mediated rearrangements was proposed [19]. We tried to use this strategy, but the unexpected high frequency of the spontaneous loss of the endogenous engineered chromosome prevented us from implementing this approach, since normal substituted XY clones were easily obtained. Even though the reason why the exogenous chromosome was lost in the first E14TG2a-based experiment but retained in the second one is unclear, we might speculate that the presence of the CEC plasmid on the pericentromeric region of the endogenous X chromosome could have, in some way, favored the elimination of the endogenous one [20].

In the present paper we provide the proof-of-principle of the chromosome transplantation approach. This “genomic therapy” might become a reality in selected situations as suggested by the experiments discussed above on trisomic cells [11] as well as by the recent report of the elimination of a ring chromosome in human cells [21]. The X chromosome was the most obvious candidate for several reasons. First, human X chromosome mosaicisms are relatively frequent; second, in differentiated cells with more than one X chromosome, only one is functional, suggesting that no gene dosage constraint exists in retaining the additional one(s); third, a number of human pathologies are located on the X chromosome. Interestingly, many of them are due to large deletions or structural abnormalities such as those often occurring at the Dystrophin locus and at the X fragile site.

Here we rescued the *Hprt* defect which in humans is responsible for the Lesch Nyhan syndrome, but with this approach various complex structural X chromosome alterations can be eliminated from the genome of iPS cells derived from affected patients, without leaving any trace of the procedure. In order to take advantage of the simple and efficient HAT selection system it would be sufficient to knockout, by nucleases such as CRISPR/Cas9, the endogenous *Hprt* locus [22] of iPS cells before applying the chromosome transplantation procedure. In this way the resulting corrected HAT-resistant transplanted cells will bear a normal diploid genome as a consequence of the introduction of the normal X chromosome followed by the loss of the endogenous one containing both the complex structural mutation and the nuclease-mediated inactivation of the *Hprt* gene. More studies are needed to show whether this approach could also be used for other chromosomes, in which an artificial selection system must be created, which would be eliminated by CRE/lox recombination. In the meantime, given the high number of X-linked disorders, the approach described here generating pluripotent cells with a transplanted or substituted chromosome adds a further layer of possible cell modifications.

## MATERIALS AND METHODS

### Cell cultures

The A9 mouse fibroblast line, *Hprt* negative derivative of strain L (Sigma-Aldrich) [23], was grown in high glucose DMEM (Lonza) supplemented with 10% fetal calf serum (FCS) (Lonza). MEFs were obtained by mincing and dissociating 13.5 days post coitum (dpc) CD-1 embryos (Charles River Laboratories) by Trypsin-Versene (Lonza). Primary cultures were maintained in high glucose DMEM medium supplemented with 10% FCS. The A9/MEF-C12 hybrid cell line was obtained by cell fusion between A9 cells and MEF cells; clones with a normal mouse X chromosome were selected in HAT (Sigma) medium and analyzed by FISH and karyotype. The cell lines were maintained by standard culture procedures in DMEM medium, supplemented with 10% FCS and HAT medium.

The *Hprt* deficient mouse embryonic stem cell lines E14TG2a and HM1 were derived from 129/Ola mice [14, 15] and purchased from ATCC.

The E14TG2a and their derivative clones were grown on 60 mm dishes coated with 0.1% gelatin (Sigma-Aldrich) in standard mouse ESC medium: Knockout DMEM (KO-DMEM; Life Technologies) containing 10% KO-Serum Replacement (Life Technologies), 1000 U/mL leukemia-inhibitory factor (ESGRO-Chemicon), 0.1 mM nonessential amino acids (Lonza), 2 mM L-glutamine (Lonza), 50 μg/ml penicillin-streptomycin (Lonza), 100 μM β-mercaptoethanol (Life Technologies); the medium was conditioned with MEFs supernatant grown for 2 days in exponential phase.

The HM1 and their derivative hybrid cells were cultured on mitomycin-C (Sigma) treated MEF feeder layer in standard mouse ESC medium using ESC-qualified FCS (Life Technologies) instead of KO-serum replacement.

### Whole cell fusion and clone isolation

A total suspension of 2×10^6^ MEF and A9 cells (1:1), were mixed together. After centrifugation at 160xg, 1 ml of a pre-warmed solution of 50% PEG (Roche) was poured onto the cell pellet over 1 min and mixed for two minutes. 10 ml of fresh complete medium was then gradually added to the cell suspension over 10 min and distributed in three 100 mm dishes. The cells were maintained in non selective medium for 24 h and then plated into ten 100 mm dishes in selective medium containing 1X HAT. Hybrid resistant clones were picked individually, culture expanded and cytogenetically analysed. A clone (A9/MEF-C12) containing the normal X chromosome was used as donor cell line in the MMCT protocol.

### Electroporation

A total of 1×10^7^ E14tg2a cells were electroporated (single pulse-250 V-path length 0.4 cm-500μF) with 10 μg of ScaI linearized CEC (chromosome elimination cassette) plasmid vector kindly supplied by T. Tada (Institute for Frontier Medical Sciences, Kyoto University, Japan) using a Pulser XCell (BioRad). Drug selection (500 ng/ml puromycin) was added after 48 h, and resistant clones were picked after 9 days.

### MMCT and clone isolation

MMCT from donor cells to recipient unsynchronised mouse ESC was performed as previously described with minor modifications [24]. In brief, mouse A9/MEF-C12 hybrid cells were used as source of microcells for the MMCT. To promote micronuclei formation the cells were treated with 0.06 μg/ml of colcemid (KaryoMAX, Life Technologies). After 48 h of incubation the cells were trypsinized, centrifuged, and the pellet was suspended in a gradient mixture containing Percoll (GE Healthcare), DMEM (1:1 v/v) and cytochalasin B (10 μg/ml, Sigma). After centrifugation at 16.000 x g for 70 min at 37°C, the isolated microcells were resuspended in 12 ml of serum-free DMEM and filtered sequentially through 8 μm and 5 μm (Millipore). The purified microcells were collected by centrifugation at 400 x g for 10 min, resuspended in 2 ml of serum-free DMEM and then mixed with an equal amount of monodispersed mouse ESCs. After centrifugation at 160 x g, 1 ml of a pre-warmed solution of 50% PEG (Roche) was poured onto the cell pellet over 1 min, followed by extensive washing in serum free DMEM. The cells were maintained in nonselective medium for 24 h, then trypsinized and split into ten 60 mm dishes containing mouse ESC medium supplemented with 1X HAT.

### Chromosome preparation and analysis

Chromosome analysis was carried out on slide preparations of cell suspensions. Briefly, cell cultures were treated with KaryoMAX at a final concentration of 0.1 μg/ml for 2 h at 37°C and mitoses were collected. After hypotonic treatment and fixation in methanol:acetic acid (3:1 v/v), the cell suspension was dropped onto a slide and air dried. Chromosome counts and karyotype analyses were done on metaphases stained with Vectashield mounting medium with DAPI (Vector Laboratories) for G-banding.

Images were captured using an Olympus BX61 Research Microscope equipped with a cooled CCD camera and analyzed with Applied Imaging Software CytoVision (CytoVision Master System with mouse karyotyping).

### Flow cytometry

Cells were prepared according to the standard protocol and re-suspended in 2% FBS/PBS on ice before flow cytometry. FACS Canto II flow cytometer equipped with Diva software (BD) were used for data acquisition.

### FISH

Whole chromosome painting probes, specific for the two sex (X and Y) and for the 18 chromosomes, the RP23-113K2 BAC (Children's Hospital, Oakland, CA) mapping to the distal region of the X chromosome, and the CEC plasmid vector were used as DNA probes. BAC and vector DNA probes were labeled *via* nick translation (Life Technologies), using biotin-16-dUTP (Roche). Flow-sorted DNA for X, Y and 18 chromosomes (M.A. Ferguson-Smith, University of Cambridge, Cambridge, UK) were labeled *via* PCR with Spectrum Orange-dUTP or Green-dUTP (Vysis).

The labelled probes were re-suspended in hybridization buffer (50% formamide, 10% dextran sulphate, 1x Denhart's solution, 0.1% SDS, 40 mM Na_2_HPO_4_ pH 6.8, 2xSSC) containing 10X mouse Cot1 DNA (Life Technologies) and denatured at 80°C for 10 min. *In situ* hybridization was performed as previously described [25]. In brief, slides were treated with Pepsin (0.004%) at 37°C for 30 sec and dehydrated through the ethanol series before denaturation in 70% formamide/2xSSC. Hybridization was completed overnight at 37°C. Stringent washes were carried out in 50% formamide/2xSSC at 42°C.

For biotin detection the slides were incubated with FITC-conjugated avidin DCS (Molecular Probes, Life Technologies), then with biotin-conjugated anti avidin D antibody (Vector Laboratories) and finally with FITC-conjugated avidin DCS. Avidin and all the antibodies were used at a final concentration of 5 μg/ml.

Slides were mounted in Vectashield mounting medium with DAPI and then were scored under an Olympus BX61 Research Microscope equipped with a cooled CCD camera. Images were captured and analyzed with Applied Imaging Software CytoVision (CytoVision Master System with Karyotyping & FISH).

### Multicolor FISH (M-FISH)

M-FISH was performed using SKY paint probe mixtures for the mouse (Applied Spectral Imaging) according to the manufacturer's protocol. Slides were mounted in Vectashield mounting medium with DAPI and then were scored under an Olympus BX61 Research Microscope equipped with a cooled CCD camera. Images were captured and analyzed with Applied Imaging Software CytoVision (CytoVision Master System with mFISH).

### Embryoid bodies

A total of 400 cells of E14TG2a, HM1 or their derivative hybrid ESC clones were cultivated in hanging drops (30 μl) on covers of 120 mm hydrophobic dishes in differentiation medium (ESC medium without LIF). After a three-day incubation, the drops were transferred to the bottom of the plates with the addition of 1 ml medium and further incubated for five days. The nascent embryoid bodies (EBs) were plated separately onto gelatin-coated 24-microwell plates.

### Immunofluorescent staining

For immunostainings, samples (mouse ESCs and EBs) were fixed in 4% PFA for 10 min at rom temperature, washed with PBS, and permeabilized in 0.3% triton X-100 in PBS for 10 min at room temperature. Primary antibodies used were *anti-Oct4* (Abcam, ab18976), *anti-Nanog* (Novus Biologicals, NB100-58842), *anti-Sox2* (Abcam, ab97959), *anti-SSEA1* (Cell signaling Technologies, MC480), *anti-Nestin* (Abcam, ab11306), *anti-Smooth Muscle Antibody* (Abcam, ab5694), *anti-Alpha-fetoprotein* (R&D System, MAB1368). After overnight primary antibody incubation, samples were washed with PBS and incubated with secondary Alexa Fluor^®^488-coniugated antibodies (Life technologies), diluted 1:2000. Samples were also counterstained with DAPI, 200 μg/ml. Slides were observed using an Olympus BX61 Research Microscope equipped with a cooled CCD camera. Images were captured and analyzed with Applied Imaging Software CytoVision.

### Sample preparation and genomic PCR analysis

Genomic DNA was extracted from cell lines using GenElute™ Mammalian Genomic DNA Miniprep Kit (Sigma) according to manufacturer's recommendations. The primer pairs and annealing temperatures are listed in Table 1 Supplemental.

PCR reactions were performed under the following conditions: initial denaturing for 5 min at 94°C; denaturing for 30 sec at 94°C, annealing temperature for 30 sec, extension for 30 sec at 72°C, repeated 35 times; final extension 5 min at 72°C. The PCR products were recovered using Wizard SV Gel and PCR Clean-Up System (Promega) and sequenced with specific primers.

### Mouse diversity array and bioinformatics analysis

For the Mouse Diversity Array [26] DNA of hybrid clones and parental cells were extracted by a conventional phenol-chloroform method. The Complete genotype using Mouse Diversity Array was performed by The Jackson Laboratory.

SNP and copy number analysis was performed using the Affymetrix JAX Mouse Diversity Genotyping Array. The array assays approximately 620,000 highly polymorphic SNPs across the mouse genome, and 900,000 invariant genomic regions used for copy number detection.

The R MouseDivGeno (1.0.4) software package [27], specifically designed to genotype the Mouse Diversity Genotyping Array, was used to analyze the files generated from this experiment together with an additional set of 249 high-quality arrays generated with the same platform as the allele calls for the samples are vastly improved when analyzed with additional high-quality data. The SNP diversity between a clone and its parental cell line has been computed as the fraction of SNPs with different genotype calls.

CNVs were identified using the ‘simpleCNV’ function from the MouseDivGeno package which integrates normalized intensities from SNP and invariant probes and uses Hidden Markov Models to infer the most likely CNV states.

### Reverse transcription-PCR (RT-PCR)

First-strand cDNAs were synthesized directly from mouse ESC and from mechanically disaggregated EBs using the Superscript^TM^CellsDirect cDNA Syntesis Kit (Life Technologies) according to the supplier protocol. PCRs were performed using specific primer pairs (Table 2 Supplemental). PCR reactions were performed under the following conditions: initial denaturing for 5 min at 94°C; denaturing for 30 sec at 94°C, annealing for 30 sec at 58°C, extension for 30 sec at 72°C, repeated 30 times; final extension for 5 min at 72°C.

### Animal use and care

The experimental facility was maintained at 23°C (± 0.5°C). The light cycle was set at 14/10 h (light/dark). Animals were given ad libitum access to food and water. The experimental procedures were carried out in agreement with Italian regulations (D.L.n.116, G.U., suppl. 40, 18 febbraio 1992, Circolare No. 8, G.U. 14 luglio 1994D.Lgs. 26/2014) and EU Directive guidelines (2010/63/EU).

### Teratoma formation and histology

To produce teratomas, 2×10^6^ mouse ESCs were inoculated subcutaneously into the flank of six-week-old CD-1 (ICR)-nu mice (Charles River Laboratories). After approximately 4 weeks resected teratomas were fixed in formalin, processed for paraffin embedding and then stained with hematoxylin and eosin (H&E).

### Cell reprogramming by nuclear transfer

B6D2F1 and C57BL/6N mice were purchased from Charles River Laboratories Italia (Calco, Italy). CD1 mice were obtained from the breeding colony of the Centro Ricerche Biotecnologiche of the Università Cattolica del Sacro Cuore (Cremona, Italy). All chemicals were supplied by Sigma-Aldrich.

Cumulus oocytes complexes (COCs) were recovered at metaphase II stage from oviducts of 7-24 week-old B6D2F1 females that had been superovulated by intraperitoneal injections of 5 IU of PMSG and 5 IU of hCG (Folligon and Chorulon, Intervet Italia) given 48 h apart. The cumulus cells were removed with 0.3% hyaluronidase in homemade HEPES-buffered KSOM (H-KSOM), and the zona pellucida was digested with 0.5% pronase in PBS. HM1-1B12 ESCs were cultured in standard mouse ESC medium as reported above and in standard mouse ESC medium supplemented with 1 μM PD0325901 and 3 μM CHIR99021 (Stemgent, San Diego, CA, USA) starting from 24 h before nuclear transfer, and were synchronized at M phase with 3 ng/ml nocodazole for 3 h prior to fusion with enucleated oocytes. Only cells of spherical shape were selected for NT. Metaphase II chromosome plates were removed by micromanipulation in H-KSOM with 5 μg/ml cytochalasin B and 10% FCS using differential interference contrast (Nikon, Eclipse TE300). After enucleation zona-free cytoplasts were individually washed for few seconds in 500 μg/ml phytohemagglutinin P in PBS and then quickly dropped over a single donor cell [28] settled at the bottom of a microdrop of the diluted donor cell suspension in H-KSOM with nocodazole. Formed cell couples were washed in 0.3 M mannitol (Ca^2+^-free, 100 μM Mg^2+^) solution and fused by double DC-pulse of 1.2 Kv/cm applied for 30 μsec. Following 10 to 15 min incubation in H-KSOM the fusion was assessed and repeated if the constructs were non fused. Cloned embryos were activated in 1 mM SrCl_2_ in Ca^2^+-free KSOM medium in 2 μl microdrops under mineral oil for 2.5-6 h and cultured in 20 μl KSOM droplets using WOW method [29] under mineral oil at 37°C and 5% CO_2_.

### Derivation and characterization of mouse NT embryonic stem cells

At day 4, blastocysts derived from nuclear transfer were co-cultured in individual wells of a 96-well feeder-coated plate in KOSR ES medium (KnockOut DMEM supplemented with 15% KOSR, 50 μg/ml gentamycin, 2 mM L-glutamine, 0.1 mM NEAA, 0.1 mM 2-mercaptoethanol, 10^3^ IU of LIF, 1 μM PD0325901, 3 μM CHIR99021 and 10 μM ACTH 1-24) for 7-11 days. Proliferating outgrowths were dissociated by trypsin and replated on feeder plates in standard mouse ESC medium with FCS until stable cell lines grew out.

### Blastocyst injection

HM1-1B12, HM1-1B36 and the NT-E3.2 were cultured in standard mouse ESC medium supplemented with 1 μM PD0325901 and 3 μM CHIR99021 starting from 24 h before injection. Morulae were recovered at 2.5 dpc from the uteri of 8-week-old superovulated C57BL/6N females, washed in H-KSOM medium and cultured in homemade KSOM medium at 37°C and 5% CO_2_ for 20 h or until blastocysts were fully expanded. Before injection cells were treated as described by Nagy *et al*. [30] and resuspended in injection medium (standard mouse ESC medium supplemented with 20 mM HEPES). Injections (5-10 cells per blastocyst) were carried out in a microdrop of injection medium under mineral oil. After injection, blastocysts were allowed to recover for 30 min at 37°C and 5% CO_2_ in KSOM medium. The resulting embryos were reimplanted (10-12 blastocysts) into a single horn of the uterus of a 2.5 dpc CD1 pseudopregnant recipient female.

## SUPPLEMENTARY MATERIAL FIGURES AND TABLES


