# Supplementary material for: Chromosome transplantation as a novel approach for correcting complex genomic disorders
Source: Oncotarget. 2015 Oct 17;6(34):35218–30. doi: 10.18632/oncotarget.6143 (PMC4742100; doi:10.18632/oncotarget.6143)
Supplement: Supplementary file 1 [file oncotarget-06-35218-s001.pdf]

Chromosome transplantation as a novel approach for correcting complex genomic disorders

Supplementary Material

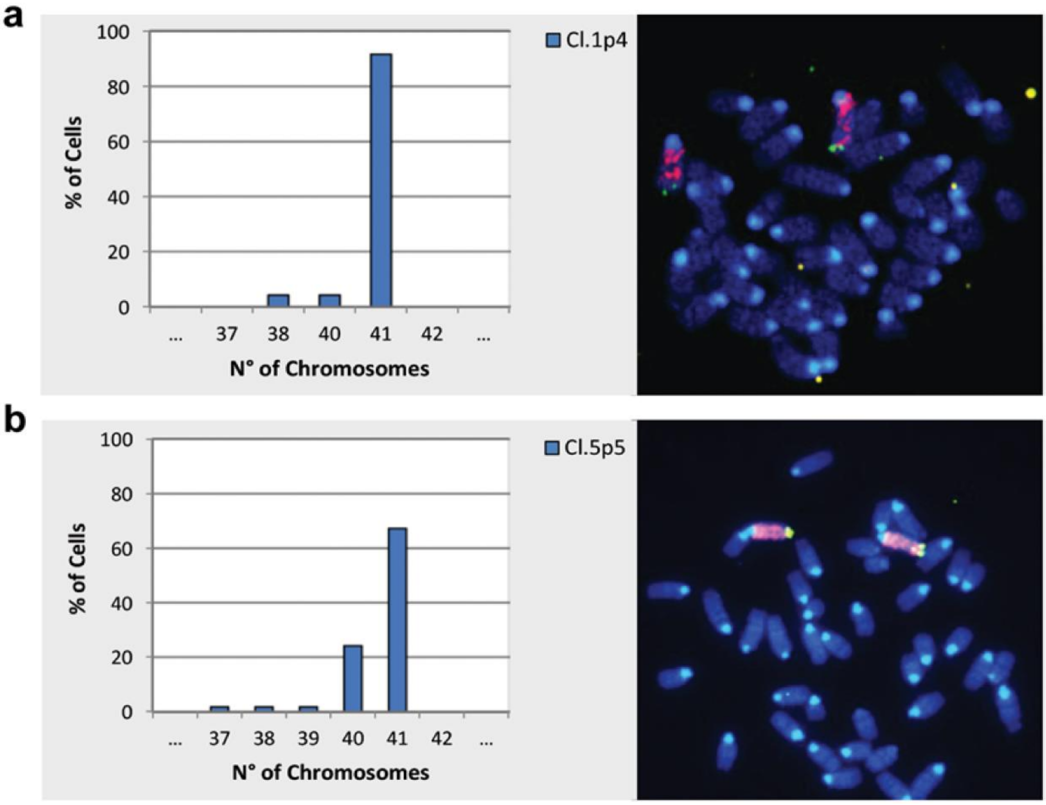

Supplemental Figure 1: Cytogenetic analysis of HAT-resistant E14TG2a-derived clones after MMCT. (**a,b**) Left: Chromosome number distributions; right: FISH metaphase spreads of the two clones. Whole mouse X chromosome painting (red) and X-linked BAC-RP23-113K2 (green) were used as probes. (n=50 each). All passage numbers are indicated with p.

a

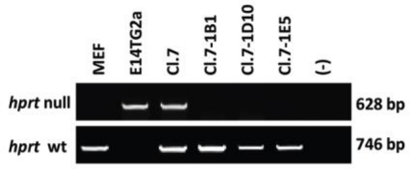

b

SNP / Clone	ChrX-70963328			ChrX-131081377	ChrX-131108926		ChrX-131126015
	rs3166628			rs8251080	rs8251054		rs8237198
E14TG2a	C	T	G	C	T	A	A
MEF	A	A	C	C/G	C/T	A/G	A/G
Cl.7	A/C	A/T	C/G	C/G	C/T	A/G	A/G
Cl.7-1B1	A	A	C	G	C	G	G
Cl.7-1D10	A	A	C	G	C	G	G
Cl.7-1E5	A	A	C	G	C	G	G

Supplemental Figure 2: Molecular analysis of the tXY subclones. (**a**) Genomic PCR of *Hprt* gene on the indicated clones. E14TG2a is the positive control of the mutated (null) locus; MEF is the positive control of the wild type (wt) locus. (**b**) Analysis of SNPs mapped to the X chromosome performed on the indicated samples.

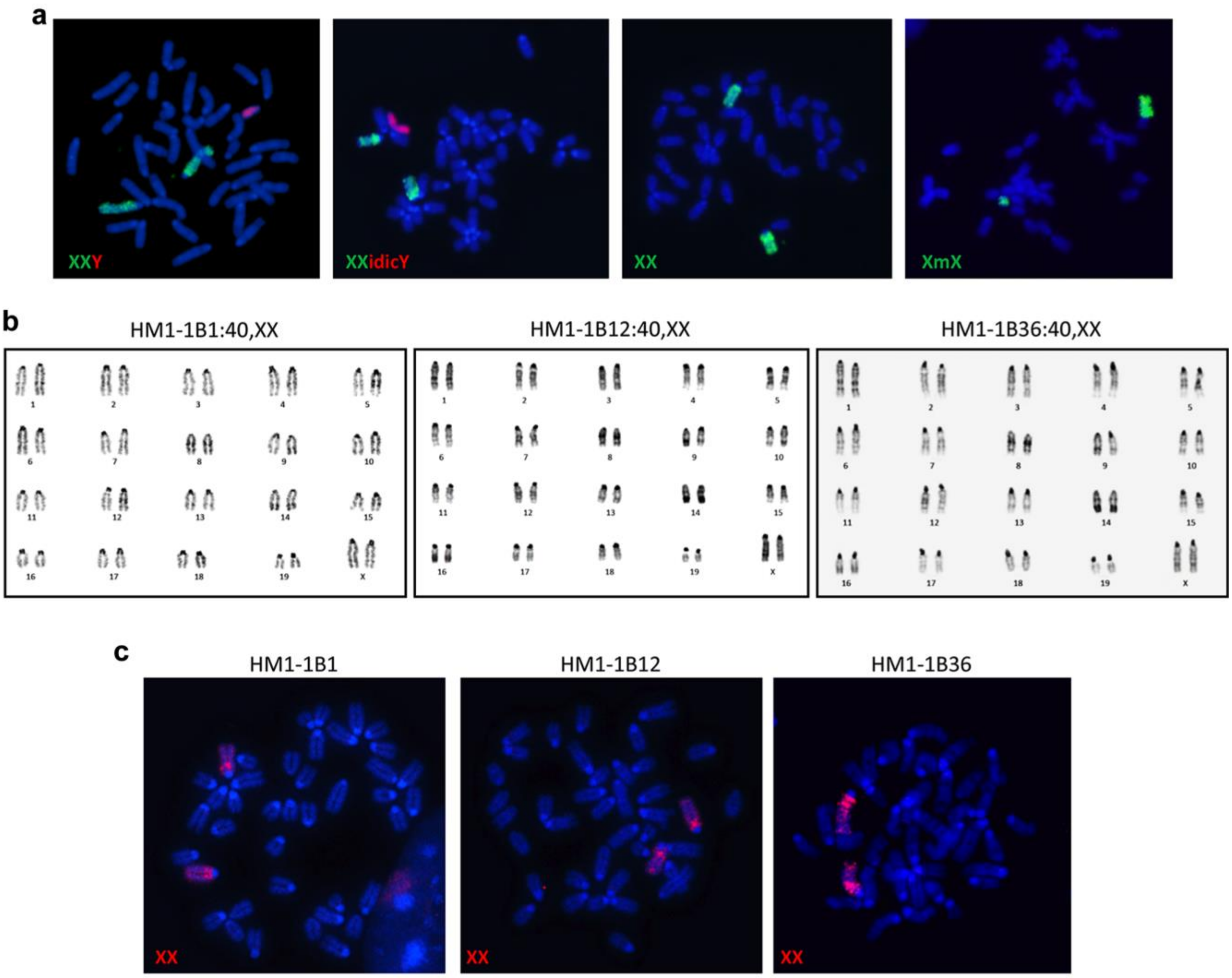

Supplemental Figure 3: Cytogenetic analysis of the sXX subclones. (**a**) Representative FISH metaphase spreads of the most frequent populations of the HM1-1B clone. Whole mouse X chromosome painting (green) and Y chromosome painting (red) were used as probes. idicY: isodicentric Y chromosome; mX: X chromosome marker. (**b**) Representative inverted DAPI-banded karyotypes. (**c**) Representative FISH metaphase spreads. Whole mouse X chromosome painting (red) was used as probe.

a

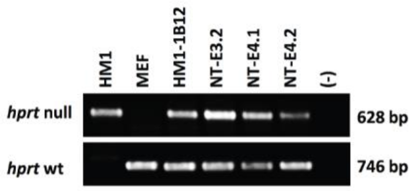

b

SNP / Clone	ChrX-70963328			ChrX-131081377	ChrX-131108926		ChrX-131126015
	rs3166628			rs8251080	rs8251054		rs8237198
HM1	C	T	G	C	T	A	A
MEF	A	A	C	C/G	C/T	A/G	A/G
HM1-1B12	A/C	A/T	C/G	C/G	C/T	A/G	A/G
NT-E3.2	A/C	A/T	C/G	C/G	C/T	A/G	A/G
NT-E4.1	A/C	A/T	C/G	C/G	C/T	A/G	A/G
NT-E4.2	A/C	A/T	C/G	C/G	C/T	A/G	A/G

Supplemental Figure 4: Molecular analysis of the rejuvenated NT-clones. (**a**) Genomic PCR detecting both forms of the *Hprt* locus on the indicated samples. HM1 is the positive control of the mutated (null) locus; MEF is the positive control of the wt locus; HM1-1B12 is the positive control of both loci. (**b**) Analysis of SNPs mapped to the X chromosome performed on the rejuvenated NT-clones.

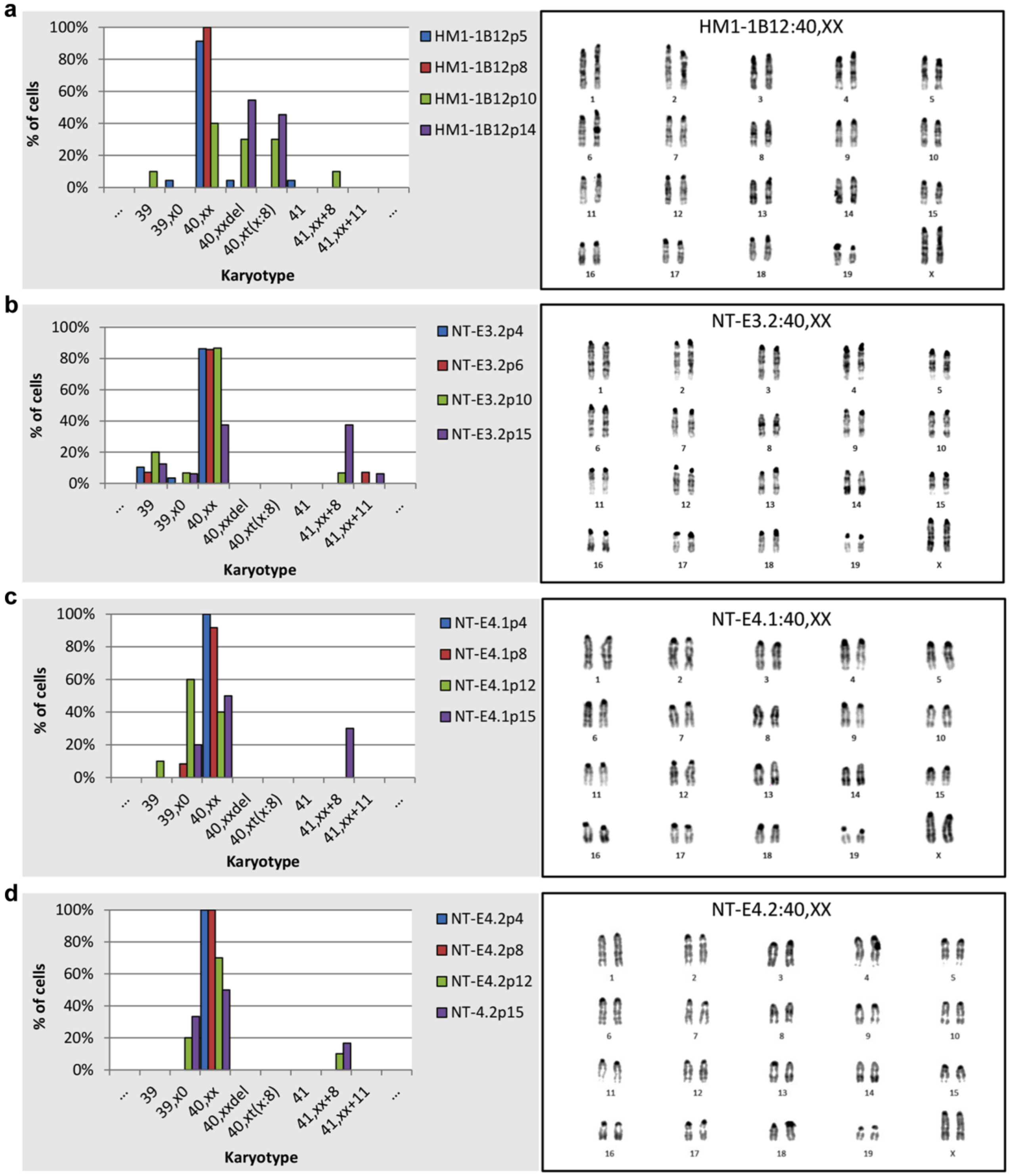

Supplemental Figure 5: Cytogenetic analysis of the HM1-1B12 subclone and its derivative rejuvenated NT-clones. (**a**) On the left, frequency of the karyotypes of the HM1-1B12 subclone at various passages; on the right, a representative karyotype of the most frequent cell population (inverted DAPI-banded). (**b,c,d**) On the left, frequency of the karyotypes and on the right a representative karyotype of the most frequent cell population (inverted DAPI-banded) of the indicated NT-clones. (n= 20 each). All passage numbers are indicated with p.

Table S1

Primers for genomic PCR			
**Name**	**Primer Sequence**	**Amplicon Size**	**Annealing T**
rs3166628	F:5'-TACATGCACTCTCGAGTGGT-3'	261bp	55□C
	R:5'-CCAATGGTGCCATTTCTCCA-3'		
rs8251054	F:5'-TCTGCCTGAGCTCCTTCTTC-3'	200bp	54□C
	F:5'-AGTTCAATTCCCAGCAACCA-3'		
rs8237198	F:5'-GGAGTCTAACGCCATTGTCC-3'	201bp	55°C
	R:5'-TGTACTTCATGGCCTGCTGA-3'		
rs8251080	F:5'-AGGCATTGACTAGCCAGCAG-3'	295bp	55□C
	R:5'-AGCCATATTGGCATCCTCAC-3'		
mSRY	F:5'-TGCTCCTGGTATGGTGTATG-3'	863bp	58°C
	R:5'-CTGTACAACCTTCTGCAGTG-3'		

Supplemental Table 1. Primers for genomic PCR.

Table S2

Primers for RT-PCR				
**Name**	**Accession N**	**Primer Sequence**	**Amplicon Size**	**Annealing T**
Nestin	NM_016701	F:5'-AGGAACCAAAAGAGGCAGGT-3'	230 bp	58°C
		R:5'-CTTGGGACCAGGGACTGTTA-3'		
Bmp2	NM_007553	F:5'-GCTCCACAAACGAGAAAAGC-3'	178 bp	58°C
		R:5'-AGCAAGGGGAAAAGGACACT-3'		
AFP	NM_007423	F:5'-ATGAAGCAAGCCCTGTGAAC-3'	158 bp	58°C
		R:5'-AGCTTGGCACAGATCCTTGT-3'		
GAPDH	NM_008084	F:5'-TGTCAGCAATGCATCCTGCA-3'	196bp	58°C
		R:5'-TGGATGCAGGGATGATGTTC-3'		

Supplemental Table 2. Primers for Real Time PCR.
